# A Remedy for Vomiting

**Published:** 1875-05

**Authors:** 


### A REMEDY FOR VOMITING.

VOMITING in Cholera Morbus and in "billious attacks" is an effort of the stomach to rid itself of that which is offensive in quality or quantity. Thus far the operation is natural and salutary. It should be promoted by drinking freely of warm water. The water should be just at that nauseating temperature known as "luke-warm." It is then a very excellent emetic. It dilutes the irritating contents of the stomach, and renders the efforts at vomiting more effectual and less distressing. It *cleanses* the stomach, and in these cases this is just what is needed.

But when the patient has already vomited freely and is suffering from ineffectual retching. then a tea cup of warm water often soothes the irritated stomach and affords permanent relief. If then the drinking of the warm water is followed, not by copious vomiting but by a period of quiet and rest, we may conclude that the cleaning out of the stomach has been thoroughly accomplished. After an hour the patient may take some mild liquid nutriment in very small quantities.

But it often happens in these cases, and also in the course of many diseases, that there is a condition of extreme irritability of the stomach which causes it to reject everything in the way of food, drink or medicine.

We have a remedy for this very troublesome complaint; a remedy not infallible, but one which has been found successful in a great many instances.

A tea cup full of corn-meal may be roasted or parched, in a frying pan, on the stove or over hot coals. The meal must be constantly stirred to prevent burning and to ensure a uniform browning to about the color of ground coffee. The roasted meal is put into a pitcher, or other convenient vessel, and a pint of boiling hot water poured upon it. The whole is then well shaken or stirred with a spoon, and allowed to settle. The clear liquid is then poured off and strained so that the patient shall not get a particle of the meal, or "grounds." Of this take a table-spoonful, warm ; or even less than a table-spoonful in cases where the stomach is very irritable. In the more common cases, half a tea cup full may be taken at first. The dose can be repeated as found necessary. This infusion of parched meal, or *corn coffee* as we may call it, is a very palatable, nutritive and refreshing beverage, used cold or warm, for persons with over-sensitive stomachs. It may be taken clear, or with cream and milk and sugar to suit the taste.

